# Signal Perception in Two-Dimensional Mapping Techniques: Just Noticeable Difference as a Visual Limit of Detection

**DOI:** 10.1021/acs.analchem.5c02398

**Published:** 2025-09-15

**Authors:** Filip Cernatič, Lukas Brunnbauer, Kristina Mervič, Jakob Willner, Andreas Limbeck, Martin Šala

**Affiliations:** † Department of Analytical Chemistry, National Institute of Chemistry, Ljubljana SI-1000, Slovenia; ‡ 27259 TU Wien, Institute of Chemical Technologies and Analytics, Vienna AT-1060, Austria; § National Institute of Chemistry, Department of Catalysis and Chemical Reaction Engineering, Ljubljana SI-1000, Slovenia

## Abstract

The limit of detection (LOD) is a ubiquitous figure of merit for characterizing the performance of instrumental methods in analytical chemistry, with the well-known formula “three times the standard deviation of the blank” serving as a common heuristic for assessing signal detection. However, in two-dimensional (2D) data settings from elemental imaging and mapping techniques, signals below the LOD often remain visually discernible. Inspired by the theory of psychophysics, we propose the Just-Noticeable Difference (JND) as a novel figure of merit for chemical data analysis in 2D contexts. The JND refers to the smallest perceptible difference between two stimuli by the human senses. By utilizing the JND as a guiding principle in targeting low-contrast signals, we offer an alternative approach to understanding detection limits in 2D data sets, with enhanced sensitivity for a large variety of sizes of spatially resolved signal and noise levels. The potential of this approach, which is presented in two different mapping techniques, LA-ICP-MS and LIBS, is compared to the standard LOD metric, which hints at the possibility for more accurate assessments of elemental concentrations and better utilization of contrast variations and spatial information inherent in mapping techniques.

## Graphical abstract

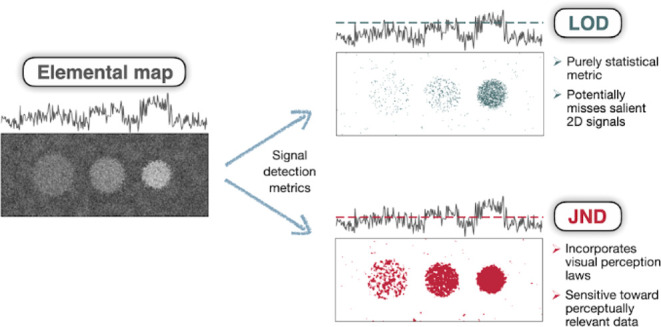

## Introduction

In analytical chemistry, the Limit of Detection (LOD) is a widely accepted metric for evaluating a given chemical measurement process. Informally, the LOD is the lowest analyte concentration at which the presence of the analyte can be confirmed with a reasonably high level of confidence. The precise working equation for LOD, which depends on the desired confidence level for determining the threshold between analyte “detected” and “not detected,” is based on a set of statistical assumptions that are not universally settled among different research institutions and regulatory bodies worldwide. According to a well-established conventional formula by IUPAC,^1^ the LOD is defined as the value of the signal that is equal to the mean (x̅_b_) plus 3.29 times the standard deviation of the blank measurements (s_b_),

1 $$\mathrm{LOD}={\mathrm{x̅}}_{b}+3.29s_{b}$$

or the corresponding concentration (in μg/g, ng/g, etc.) which produces that signal. In practice, particularly when the blanks are not readily available, the LOD concentration value is often estimated from (univariate) calibration data as $c_{LOD}=3.29\;*\;s_{y/x}/k$ , where $s_{y/x}$ is the residual standard deviation of the calibration, and *k* is the slope of the calibration curve.^2^ The choice of the prefactor 3.29 in the above definition rests on the assumption of normally distributed measurements with constant variances in the entire concentration range (homoscedasticity) and accounts for the 5% probability of both Type I error (i.e., making the decision “analyte detected” when there is no analyte in the sample) and Type II error (failing to detect an analyte that is present in the sample). The choice of 5% is, in principle, arbitrary, as is the assumption of normality, and both constraints can be changed in certain scenarios, depending on the number of samples and the differences in practical costs of the two types of errors. In this sense, the prefactor 3.29 can be understood as a trade-off that is acceptable for most analytical methods. If *s*_*b*_ is estimated from a limited set of measurements, then IUPAC recommends the use of Student’s *t* distribution in determining the correct prefactor given the desired confidence level,^1^ as usual for small samples. Apart from IUPAC, other guidelines exist, such as the European Commission^3^ or the Food and Drug Administration guideline,^4^ which employ slightly different statistical criteria for calculating the LOD. In any case, LOD is a generally applicable metric, regardless of the analyte of interest, and the instrumental method used in the chemical measurement process. Apart from the aforementioned calibration approach, which can be formally described as a collection of zero-dimensional (0D) concentration-signal data points, LOD can also be determined directly from experimental measurements on real samples, which is particularly common in methods that report the data as time series or one-dimensional (1D) plots, such as chromatographic, electrochemical, and spectroscopic methods.^5,6^ In these scenarios, LOD is frequently determined from the signal-to-noise ratio (SNR or S/N), which is the ratio of the height of the peak (signal) of a measured sample compared to the standard deviation of the baseline (noise). A widely adopted heuristic criterion for reporting the LOD is to calculate the concentration at which *S*/*N* = 3. By analogy, in two-dimensional imaging data, one may determine the LOD by comparing the maximum or mean signal value of a region of interest (ROI) to the noise of its surroundings (background). One commonly used metric for signal assessment in images is the contrast-to-noise ratio (CNR).^7,8^ According to one of the definitions, which can be directly related to LOD, the CNR metric is calculated as the difference in mean numerical signal values between the ROI (x̅_ROI_) and the background (x̅_bg_), divided by the standard deviation of the background (s_bg_),^9^

2 $$\mathrm{CNR}=\frac{\left|{\mathrm{x̅}}_{\mathrm{ROI}}-{\mathrm{x̅}}_{\mathrm{bg}}\right|}{s_{\mathrm{bg}}}$$

In line with eq 1, LOD is exceeded whenever *CNR* > 3.29. A number of other metrics have been designed specifically for image analysis, such as the local signal-to-background ratio (LSBR),^10,11^ which is defined as the sum of squared differences from the mean, divided by the standard deviation in a rectangular ROI of width W and height H

3 $$\mathrm{LSBR}=10\log_{10}\left(\frac{\overset{W}{\underset{i=0}{\sum }}\overset{H}{\underset{j=0}{\sum }}{\left(x_{ij}-{\mathrm{x̅}}_{\mathrm{ROI}}\right)}^{2}}{s_{\mathrm{ROI}}^{2}}\right)$$

or the recently proposed pixel-wise signal-to-noise ratio (pwSNR),^12^ which calculates the average absolute deviation (*E*_ROI_) of signal intensities between the ROI and the mean background value, divided by the background standard deviation

4 $$\mathrm{pwSNR}=\frac{E_{\mathrm{ROI}}\left(\left|x_{ij}-{\mathrm{x̅}}_{bg}\right|\right)}{s_{bg}}$$

One strong advantage of metrics such as CNR, LSBR, and pwSNR is that they are easily interpretable functions of the means and standard deviations of the image regions under consideration. However, the LSBR and pwSNR do not lend themselves to a definitive criterion in terms of a simple threshold for signal detection, and furthermore, none of these metrics consider the crucial difference between the visual presentations of imaging data and lower-dimensional (i.e., 0D and 1D) data. In usual scientific images, which are digitally stored as arrays of numbers, different numerical values directly correspond to different color values of a predetermined color scheme, which is intended to guide the observer to focus the attention on visually relevant parts of the image and away from contentless surroundings. In this respect, two properties of human visual system, which are not considered by summary statistics alone, namely, the sensitivity of the human visual system to small color differences and the information on spatial correlations of features in two-dimensional data, are both key to detecting objects in 2D that are otherwise virtually impossible to notice in 1D settings with comparable noise levels. Concerning spatial correlations in particular, it is well known from psychophysics that the human visual system is highly adept at amplifying signals by integrating redundant information, like regions of similar brightness, when identifying meaningful structures in an image.^13,14^ Since the potential for spatial redundancy in 2D is much higher than in 1D data representations, it is reasonable to assume that objects of comparable size and signal intensity are more easily detectable in 2D than in 1D.

A striking example is illustrated in Figure 1a, which is an artificially constructed digital image, consisting of five circular features on a background with Gaussian noise of constant mean brightness and standard deviation (100 and 5, respectively, in 8-bit gray-level units). Figure 1b represents the profile of signal intensity at the hundredth line of Figure 1a. The circular spots in Figure 1a were obtained by increasing the mean of the background noise while keeping the standard deviation constant. While in the image (Figure 1a), even the leftmost spot is dimly observable in most environments, in the corresponding horizontal midline (Figure 1b), the sections in the graph with increased mean values are challenging to visually isolate (apart from the rightmost one with 3.29 sigma) without the guidance of the top image and red demarcations. This observation is in line with the guidance for signal recognition in 1D data, which is actually in Figure 1b actually is. Given that even such a simple image presents a challenge when it comes to the applicability of the LOD as an analytical figure of merit, it is not unreasonable to speculate that evaluating digital images of real data would be even more challenging. In light of this problem, we propose an alternative metric for application in 2D imaging data: an analogous concept from psychophysics, the so-called “Just-Noticeable Difference” (JND),^15^ which is the smallest difference in magnitude between two stimuli that can be observed by human senses like vision and hearing. Given an absolute intensity (brightness) of a target ROI, *I*_1_, and a reference region (usually the surroundings of a given ROI), *I*_*0*_, the just-noticeable difference is computed as JND = *I*_1_ – *I*_0_. Several laws have been proposed to mathematically model the JND, with limited applicability in different settings. For instance, Weber’s law^16^ states that the JND is linearly proportional to the intensity of the reference, i.e., JND = *kI*_0_, where *k* is an empirical constant. Weber’s law implies that visual stimuli are perceived logarithmically across the dynamic range of light intensities, which is a reasonable approximation at moderate brightness levels. In dark images or low-brightness settings, the incident photons from the observed object arriving at the eye are usually assumed to obey the Poisson counting statistics, which is embodied in the Rose model,^17,18^ which states that $\mathrm{JND}=k_{\mathrm{Rose}}\sqrt{I_{0}}$ Generally speaking though, visual perception has a highly nonlinear relationship to the intensity of a given stimulus, and a JND function that accurately reflects reality should also depend on local image properties like edge contrasts and textures, thus precluding objective description of the process by a single unified model. In general, JND is determined statistically from experiments, usually as a difference that is noticed on 50% of trials.^19^ For grayscale images, where each pixel is represented by a single number, a JND function is determined in an experimental setting by recording the subjective reports of just visible gray values of an ROI of fixed shape against reference background gray levels and fitting the reported data to a predetermined function. In the literature on computer vision and image/video coding, several approximate JND-estimation models have been proposed,^20–22^ with different functional forms, most of them consisting of a U-shaped convex function that emphasizes high sensitivity to middle gray levels, and lowered sensitivity to black and white limits.^23^ In this paper, we adopt the model of Chou and Li^20^, which is a simple piecewise JND function of background intensity (with a square root dependence for darker regions and a linear dependence for brighter regions). This model has been used in many recent studies^24–27^ in digital image processing, and we have found it to be the most suitable model for describing the sensitivity to grayscale differences when viewing images on a typical desktop computer.

**Figure 1:**
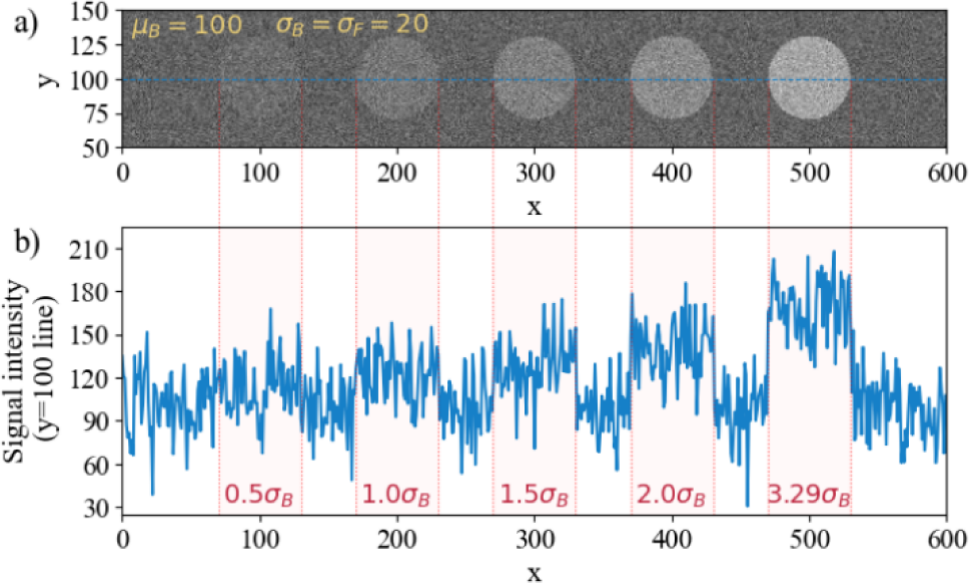
Comparison of the visibility of signals in a generic grayscale image (a) versus one-dimensional data from the horizontal line at y = 100 (b). The signal intensities of the circles and background in (a), which are displayed in 8-bit gray-level units, are drawn from a normal distribution with a variance of 5. The mean intensity value of the background is equal to 100, while the means of the five circles increase in steps from 100 to 100 + 3.29*s_b_* (IUPAC LOD threshold), with the intensity differences displayed in red at the bottom of (b).

In this work, we present an application of the proposed JND metric on elemental mapping data, i.e., spatially resolved maps from two cutting-edge instrumental chemical analysis techniques: the Laser-Induced Breakdown Spectroscopy (LIBS)^28^ and Laser Ablation Inductively-Coupled Plasma Mass Spectrometry (LA-ICP-MS).^29^ A detailed comparison is made with the LOD in terms of the calculated thresholds. We demonstrate by theoretical investigations that the combination of a simple image preprocessing scheme and the evaluation of JND based on automatically calculated image background regions can lead to a sensitive approach for clearly discerning visually detectable low-contrast signals in elemental maps that the conventional LOD metric can either struggle to pinpoint or miss signals altogether, while also reporting concentration detection limits closer to actual elemental concentrations in samples. In maps with clearer signals, we show that the JND performs just as well as the LOD in signal detection. In addition, a set of calculations of JND for different beam sizes is carried out on LA-ICP-MS data, and a summary discussion is provided on determining the optimal beam size for detecting a given object size. For the sake of simplicity, we work with 8-bit grayscale images, where a single pixel is described by a scalar variable representing brightness or luminance. In 8-bit grayscale images, the brightness level is represented by integers ranging from 0 (black) to 255 (white), with intermediate values representing different shades of gray. Other compelling reasons for choosing this bit depth include the fact that the majority of applications for viewing digital images are limited to 8-bit data, and experimental studies provide strong evidence that the human eye can discern at most about 700–900 shades of gray in higher bit-depth medical displays.^19^ Although not discussed here, the analysis of this work can be extended to grayscale images with larger bit depths by simple rescaling of the JND function, and in principle, even to colored images with full three-dimensional color spaces such as RGB, CIELAB, or YCbCr, where each pixel is represented by a vector of three color channels.^30–33^ We also stress that the developments presented here are completely general and applicable to other powerful techniques amenable to imaging or mapping, such as X-ray fluorescence (XRF) microscopy,^34,35^ secondary ion mass spectrometry (SIMS),^36^ matrix-assisted laser desorption/ionization imaging mass spectrometry (MALDI-IMS),^37,38^ electron energy loss spectroscopy (EELS),^39,40^ Raman spectroscopy^41,42^ and atomic force microscopy (AFM)^43,44^ to name a few.

## Experimental Section

### Construction of Artificial Samples

Artificial samples were prepared by using pulsed laser deposition and photolithography in combination with ion etching. The approach and instrumentation used are described in more detail by Schraknepper.^45^ In short, two different samples were prepared. For both samples, photolithography was used to create structured thin films (circles with diameters ranging from 20 to 200 μm) on a substrate material. The combinations of substrates and thin films are Al_2_O_3_/50 nm Al:STO (0.5% Al in SrTiO_3_) and YSZ/100 nm Pt:LSF (1% Pt in La_0.6_Sr_0.4_FeO_3_ on the yttria-stabilized zirconia substrate).

### LIBS Experimental Conditions

LIBS measurements were carried out using an imageGEO193 laser ablation system (ESL, Bozeman, Montana, US) operating at a wavelength of 193 nm. Light emitted from the generated plasma was collected using an optical fiber, which was connected to a high-resolution spectrometer (HRS-750-MS, Princeton Instruments) with an ICCD camera (PI MAX4, Princeton Instruments). The spectrometer was set to a center wavelength of 407 nm with a grating of 600 g/mm. Data were recorded with a gate delay of 0.1 μs and a gate width of 10 μs. For LIBS measurements, a laser energy of 1.2 J/cm^2^ and a spotsize of 5 × 5 μm^2^ were used. For the estimation of the LOD and JND in the concentration domain, calibration slopes for Fe and La were determined from average signal intensities of a small section of the largest spot in their respective elemental maps (see Supporting Information).

### LA-ICP-MS Experimental Conditions

The LA-ICP-MS experiments were carried out using an Analyte G2 193 nm ArF* excimer laser ablation system (Teledyne Photon Machines Inc., Bozeman, MT) at the National Institute of Chemistry in Ljubljana (NIC). The LA system equipped with a HelEx II standard two-volume ablation cell was coupled to a Vitesse ICP-TOF-MS (Nu Instruments, Wrexham, UK) via the Aerosol Rapid Introduction System (ARIS) from Teledyne Photon Machines. Line scans were performed on Pt:LSF on YZS and Al:STO on Al_2_O_3_ samples provided by the TU Wien. The mapping experiments were performed with three different square beam sizes—5, 10, and 20 μm—to assess spatial resolution and elemental distribution. A two-point calibration was performed using NIST SRM 610 and 612 glass standards^46^ to estimate concentrations. While these standards served as a proof of concept, they may not represent an ideal calibration match for the materials studied. The complete set of operating parameters for these experiments is summarized in Table 1.

**Table 1:** Instrumental Parameters Used for the NIC Laser Ablation System Coupled with TOF-ICP-MS

LA (Analyte G2, ARIS)	
Wavelength (nm)	193
Laser fluence (J cm^–2^)	1.0
Repetition rate (Hz)	100
Scanning mode	Line scanning
Dosage (shots per pixel)	10
Washout time (ms)	ca. 40
Beam size (μm)	5, 10, 20
Mask shape	Square
He carrier flow rate (L min^–1^) cup|cell	0.3|0.3
ICP-MS (Vitesse)	
RF power (W)	1300
Auxiliary gas flow (L min^–1^)	2
Coolant flow (L min^–1^)	13
Nebulizer flow (L min^–1^)	1.2
Reaction cell gas (mL min^–1^)	6 (He)/15 (H_2_)

## Results and Discussion

### Theoretical Modeling and Studies: Toward a New JND-Based Figure of Merit

A web-based application was developed in the Python-based Dash framework, which allows the user to import single 2D maps as csv files and perform rudimentary image preprocessing, including the clipping of outliers and normalization, a manual or automatic background selection, and calculation of LOD and JND metrics. In addition, visualization of LOD- and JND-based thresholding maps is implemented in the application, which is particularly suitable for locating features in low-contrast and/or high-noise environments. Instructions on how to use the application, as well as the details of the inbuilt image processing and the thresholding algorithms, are described exhaustively in Supporting Information. The application is accessible online via the following link: http://chem-imaging-apps.ki.si/lod-jnd-app. With the in-house application, we tested hundreds of elemental maps from LIBS and LA-ICP-MS techniques for some of the elements present in the aforementioned sample. The sensitivity of the two metrics in question (IUPAC LOD and the proposed JND) is compared in the numerical results, which are shown in Figures 2–4 and Table 2. For LOD, we used eq 1 given in the introduction, where we replaced the mean of the blank with the average value of the background, chosen by the application’s algorithm (more on that in the Supporting Information). For the JND, we propose the following formula,

$$\mathrm{JND}=x_{\mathrm{bg},0.95}^{\prime }+\mathrm{LA}\left(x_{\mathrm{bg},0.95}^{\prime }\right)$$ 5

where $x_{\mathrm{bg},0.95}^{\prime }$ is the gray level of the 95th percentile of the background intensity distribution of a modified image $I^{\prime }=I\;*\;M$ obtained via convolution of *I* with a 3 × 3 median filter *M*, and LA is the so-called luminance adaptation function from the model of Chou and Li, which reads as

6 $$\mathrm{LA}\left(x\right)=\{\begin{array}{l} 17\left(1\;-\;\sqrt{\frac{x}{127}}\right)+3,\mathrm{if}\;0\leq x\leq 127\\ \frac{3}{128}\left(x\;-\;127\right)+3,\mathrm{if}\;127<x\leq 255. \end{array}$$

**Table 2:** A Collection of Representative Signal Intensity Values, JND, and LOD Metrics from Elemental Maps in Figures 2 and 3

Representative intensity, LOD and JND values in elemental maps from Figures 2 and 3	Fe (LIBS, 20%, gain 25)	La (LIBS, 10%, gain 25)	Gd (LA-ICP-MS)	Ba (LA-ICP-MS)
Range (min – max)	6.67–80.3	3.95–31.81	0–100.09	0–83.05
Background average	35.08	11.79	0.9186	3.529
Background standard deviation	12.15	3.511	3.213	6.492
LOD	75.06	23.35	11.49	24.88
JND	44.34	14.60	7.85	11.92
LOD concentration	929.7 mg/g	623.3 mg/g	34.23 μg/g	35.25 μg/g
JND concentration	215.2 mg/g	151.4 mg/g	33.37 μg/g	34.96 μg/g

**Figure 2:**
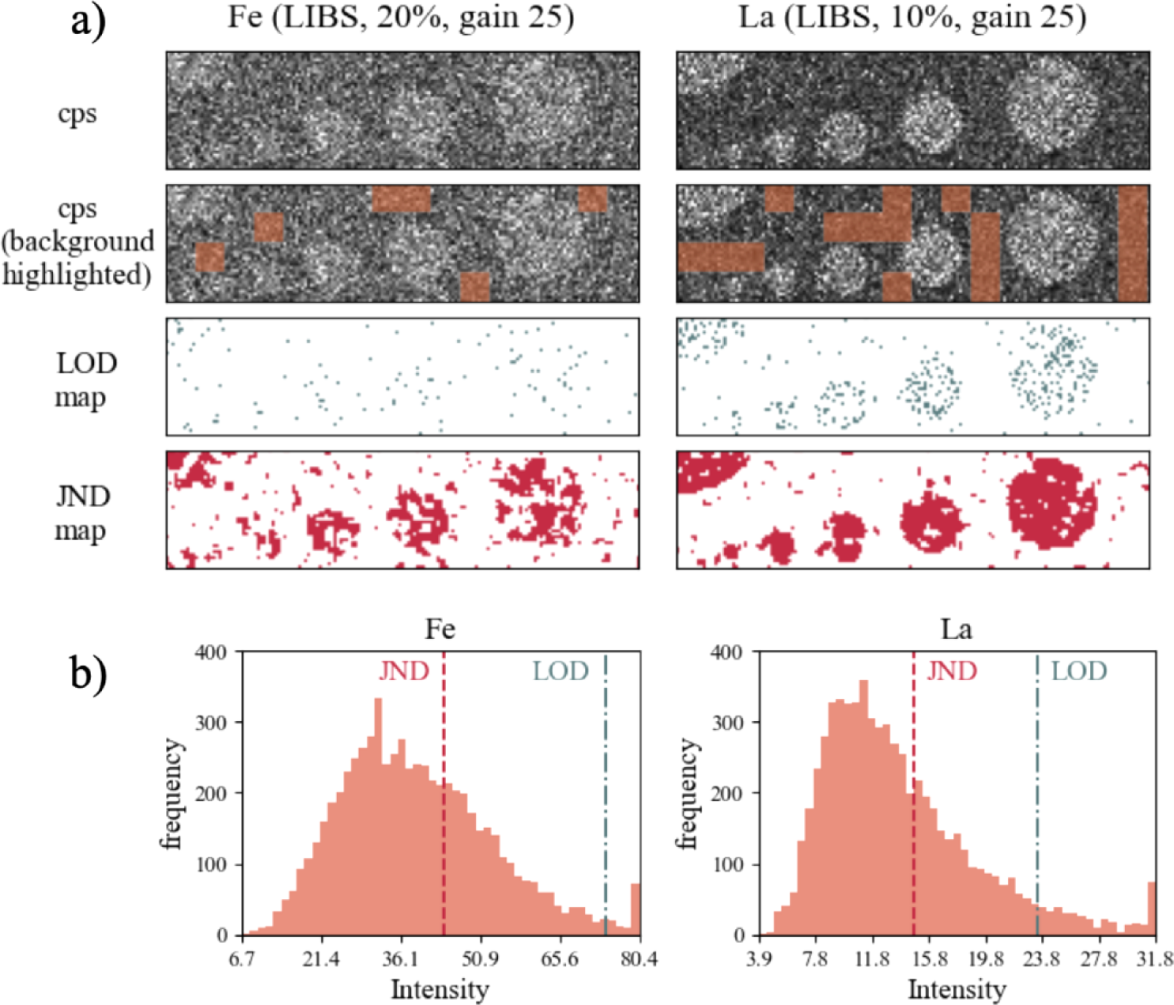
(a) A qualitative comparison between the LOD and JND metrics in terms of their ability to discern features from noise in LIBS elemental maps of the Pt:LSF system (left column, Fe; right column, La). Top: Elemental maps of absolute background-corrected signal intensities, where lighter-shade gray pixels represent high values and darker-shade gray pixels represent low values. Second from top: Elemental cps map, with background areas highlighted (as determined by the algorithm). Second to bottom: LOD map – labels all pixels with cps higher than the LOD of the background. Bottom: JND map – labels all pixels with cps higher than the JND of the background. (b) Corresponding histograms (left: Fe, right: La), with vertical lines at the JND and LOD thresholds.

**Figure 3:**
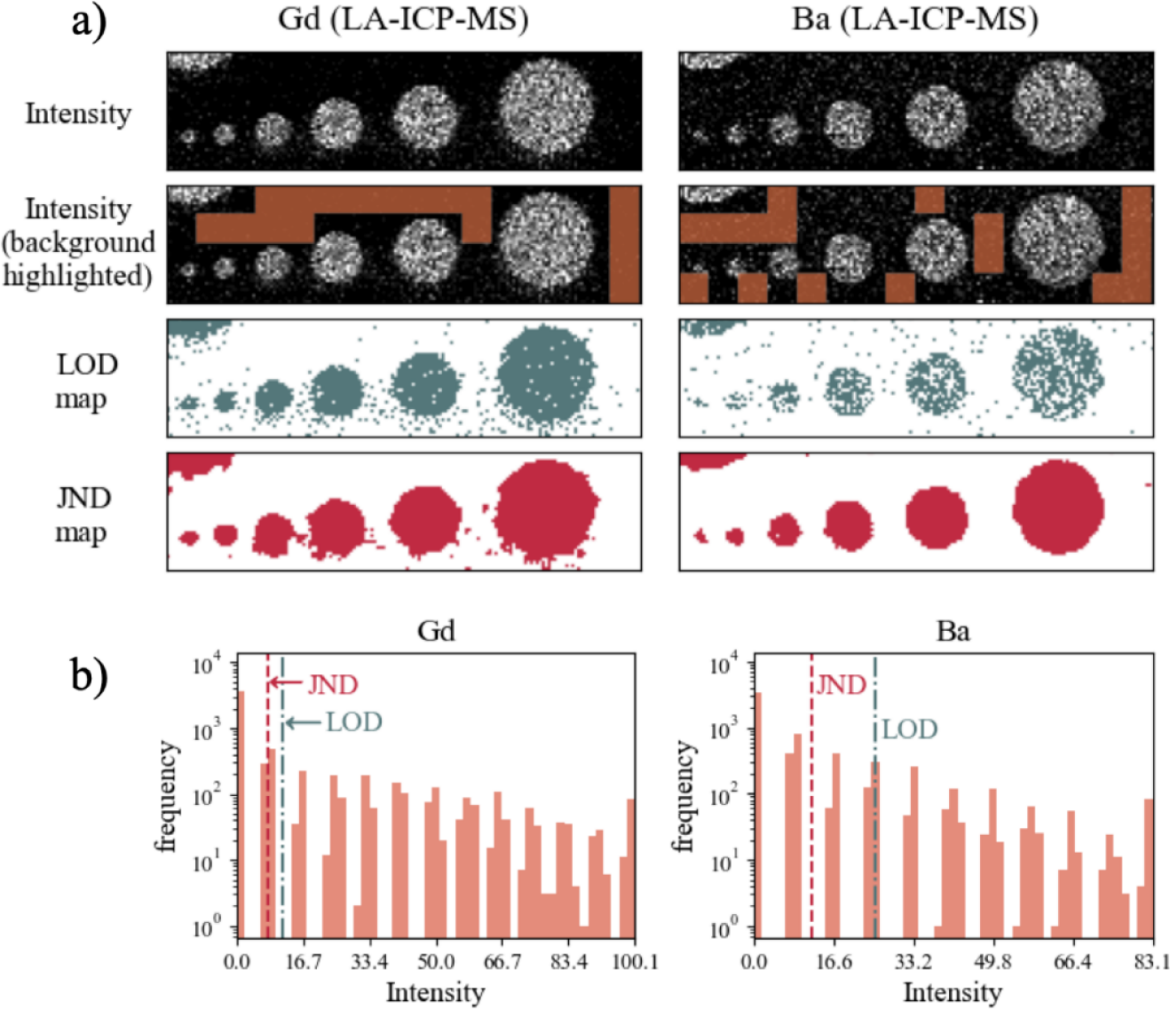
(a) An analogous comparison between the LOD and JND metrics in two examples of LA-ICP-MS elemental maps of the Pt:LSF system (left column – Gd, right column – Ba). (b) Corresponding histograms (left: Gd, right: Ba), with vertical lines for the JND and LOD thresholds.

**Figure 4:**
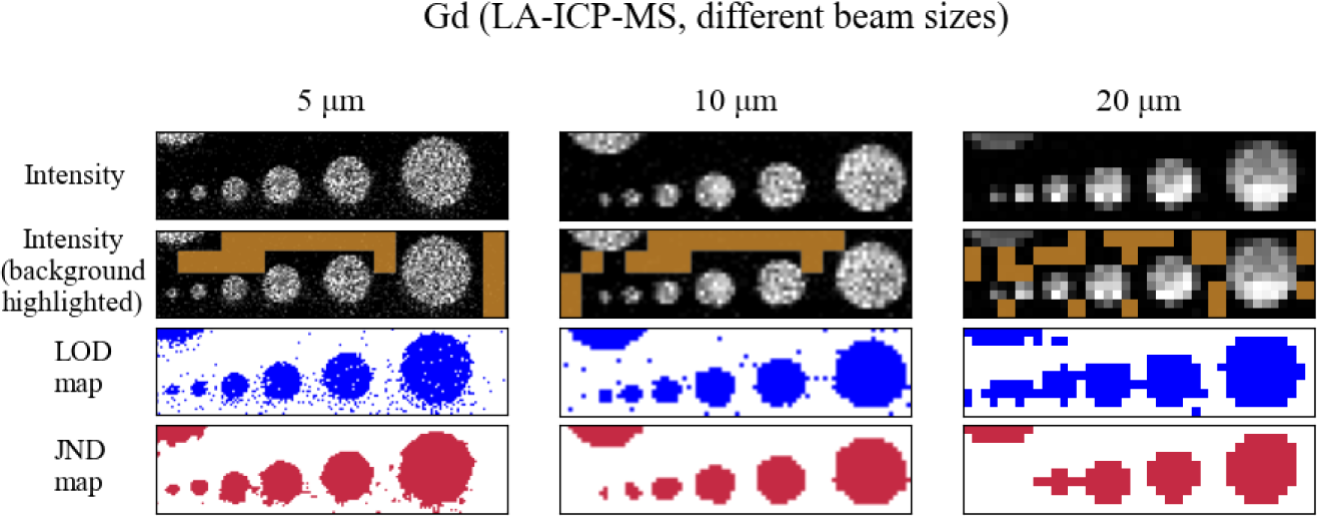
Comparison of JND maps for the Gd elemental map from LA-ICP-MS at different beam sizes.

With the background automatically selected by the application, the above function is applied to the median-filtered version of the image under study. Subsequently, all pixels that fulfill the criterion I’>JND are labeled as exceeding the just-noticeable difference. In the case of LOD, raw unfiltered data is used, so that pixels fulfilling the condition I > LOD are labeled as exceeding the conventional limit of detection. We chose the median as a measure of centrality for locally filtering pixels in images since it is a ubiquitous operation in image processing, which is more robust to outliers than the mean value, and is particularly efficient at removing the well-known “salt-and-pepper” noise^47^ arising from outlier pixels with significantly higher or lower intensities from their local surroundings. The size of the filter (3 × 3) was found to produce reasonably gentle denoising without overly smoothing local edges.

### LOD vs JND: Examples from LIBS and LA-ICP-MS

The results of theoretical investigations into the feasibility of using the standard IUPAC LOD versus our proposed JND-based metric for detection limits on four illustrative examples of elemental maps from the two instrumental methods (beam size 5 μm) are presented in Figures 2, 3 and Table 2. For the final theoretical analysis, elemental maps from the Pt:LSF system were chosen since they contained visually well-resolved features for major elements such as Fe and La (see Figure 2) as well as some other, unintended constituent elements, like Gd and Ba shown in Figure 3. All LOD and JND values are reported in intensity units corresponding to the total integrated counts for LIBS and counts per second (cps) for LA-ICP-MS. Transformation of the respective metrics to the concentration domain was carried out in different ways, depending on the instrumental method. For LA-ICP-MS maps, concentration values for LOD and JND were obtained by the aforementioned two-point calibration with NIST glass standards, while concentrations for LIBS maps were estimated from the average signal intensity of the largest spot in the sample (see Supporting Information). Since a major objective of modern instrumental methods is pushing toward detection of ever lower-intensity signals and reliable determination of trace element concentrations, we also scrutinized both LOD and JND in terms of their ability to discern salient features in elemental maps as potential signal candidates. Across the four different examples presented in Figures 2 and 3, the two metrics vary considerably in their value and hence in their abilities to detect different concentrations of elements and elucidate the sample’s circular spot features in a way that stand out from the background. Figure 2A shows two example maps from LIBS measurements with the Fe map obtained at 10% laser energy and 25 gain and the La map at 20% energy and 25 gain. In both maps, the calculated JND is substantially lower (in the order of several 10%) than LOD in both maps. In the Fe map, which contains very dimly visible low-contrast features, LOD is clearly high, positioned relatively close to the maximum intensity due to a very high-noise background with complex gray patterns (background standard deviation is about 15% of the maximum intensity). As a result, the LOD is unable to effectively distinguish any salient circular features on the image. On the contrary, the JND value is much lower, about half the maximum intensity, rendering a much larger proportion of pixels on the map than LOD and uncovering a few large connected regions, but also a high number of smaller regions that can be qualitatively determined as false positives. Since Fe intensity distribution is unimodal (see Figure 2B), a large number of potential false positive signals are expected, and a single value JND threshold may not be the most informative metric in such cases. Computing the JND metric at different background percentile levels (eq 5) and visually inspecting the resulting JND could serve as an aid in determining the optimal threshold. Nevertheless, for consistent results, we keep in our calculations the 95th percentile of the background as in eq 5. In the La map, the LOD and JND are slightly closer to each other, with the JND still being lower by about 20% of the full signal intensity range. In this case, LOD is low enough to be able to outline the circular spots slightly better than in the Fe map, except for the smallest two circles, which are much more clearly shown in the JND map. This is because the intensity distribution in the La map is more skewed toward lower values, around a 25% maximum intensity. Still, JND still shows much more pronounced clusters of pixels than LOD. Considering the two metrics in concentration units, it is instructive to compare them to actual concentrations of mapped elements in the thin films of the sample material (La_0.6_Sr_0.4_FeO_3_), which are equal to 215.99 mg/g for Fe and 375.02 mg/g for La. From Table 2, we see that the LOD is higher by hundreds of mg/g for both elements (929.7 mg/g for Fe and 623.3 mg/g for La, respectively), while the JND is lower but comparable to the elemental concentration for Fe (215.2 mg/g), and substantially lower for La (151.4 mg/g). These results suggest that JND can reliably detect elemental signals in low-contrast and noisy elemental maps, which LOD struggles to capture.

In LA-ICP-MS maps, which are shown in Figure 3, the background noise levels are comparatively lower than the noise from LIBS maps (according to Table 2, background standard deviations are on the order of a few % of the maximum intensity). The JND value for the two LA-ICP-MS maps is also lower than the LIBS JNDs, and about 8% of the maximum cps values, which is close to the JND value of the lowest intensity (which maps to the black color, according to the model by Chou and Li^20^ used in this work, JND(0) = 20 ≈ 7.84% of 255) since the background intensities are very low and mostly get mapped to dark colors. The use of median filtering also contributes to equalizing the JND since it almost completely removes single-pixel and other small isolated clusters of outliers with high intensities. On the contrary, the LOD value for Ba is noticeably higher than Gd LOD, since the average background intensity is about four times higher and the standard deviation is twice as high (Table 2) in the Ba map compared to the Gd map, which results in a considerably higher LOD value for Ba. As a result, there is a window of signal intensities, with a width of about 17% maximum intensity, in which the JND can discern potential features that the LOD cannot (see Figure 3b, Ba histogram). Concerning the distinguishability of circular features actually present in the sample, both LOD and JND yield comparable outcomes, with the exception of Ba LOD map, where the smallest two circles are difficult to visually discern from the environment. LOD essentially fails to capture the smallest two circles. Nevertheless, both LOD and JND perform comparably well since the background is relatively flat and the signals of circular spots are high with clearly delineated edges, which is not the case in the presented LIBS maps in Figure 2. The corresponding concentration values for JND are lower than LOD, but their difference is very small (less than 1 μg/g for both elements, which is below the calibration errors—NIST SRM standards have errors in 10° μg/g for Gd and Ba). This is simply a consequence of large values for the calibration slopes and the intercepts, which suppress any differences in the intensity domain. Hence, taking into consideration the comparison between calculated metrics in concentration units, the use of JND does not lead to a substantial benefit in these maps. A more accurate assessment would require comparison with actual concentrations of Gd and Ba in the thin films of the sample, which are unknown.

### A Discussion on the Appropriate Beam Size on the Resolution of Different-Sized Features

We also analyzed the effect of laser beam size (in LA-ICP-MS only) on the recorded elemental maps, with three different beam diameters: 5, 10, and 20 μm and investigated the ability of JND and LOD to detect signals. To make the maps with larger beams the same size and shape as the 5 μm maps discussed in the previous section, the aspect ratio was kept constant at 4:1. To further simplify the comparison, the Gd map from LA-ICP-MS measurements was chosen because it has a low-noise background and the most similar JND and LOD values. In Figure 4 we can observe that increasing the beam size drastically reduces the frequency of single-pixel outliers in the background (to negligible levels at 20 μm) but simultaneously blurs the boundaries of features, and the smaller circles lose their distinctive shape. Moreover, the smallest two circles in the 20 μm map are represented by clusters of three to four dark gray-level pixels, which are dropped out by the application of the 3 × 3 median filter since the surroundings are black pixels of low intensity from the background. Hence, the JND fails to detect them properly. In addition, the next two larger circles in size on the 20 μm map are detected by JND as a single unresolved feature. LOD, which is actually lower than JND (1% vs 8% of the maximum intensity, respectively) in the 20 μm map, also fails to properly discern the circles, with all but the largest one lumped together. This problem raises a question that has hitherto not been considered, namely, “What is the largest appropriate beam size that can still clearly distinguish a feature of a certain size in a given elemental map?” Assuming that the background is a simple region of a single intensity level, or a narrow distribution of similar intensities, and that features have an average intensity high enough to exceed the JND, one criterion that could be employed in the attempt to address the beam-size concern is the well-known Nyquist–Shannon Sampling theorem^48,49^ from digital signal processing theory. The theorem states that the sample rate of a continuous bandlimited signal should be at minimum twice its highest frequency component, i.e., fs ≥ 2f_max_ in order to completely reconstruct the original signal. Sampling below this criterion is known to result in a loss of details and lead to artifacts such as aliasing, which can significantly distort the original signal content. When applied to 2D signals in imaging, the theorem translates into a simple rule that dictates the sampling of “at least two pixels per smallest feature”. Some related guidelines exist in other well-established imaging methodologies, for instance, in confocal microscopy,^50^ the factor 2 is increased to vary between 2.3 and 4 to stay on the safe side of clearly determining the smallest objects. We suggest a simple rule of thumb to adjust the beam size to at least 1/3 the area-equivalent diameter of the smallest expected feature size, which implies a minimum factor of 3 in the Nyquist–Shannon theorem. This way, at least two pixels will be fully contained inside the feature (assuming a circular shape). We do have to keep in mind that images from real samples often have a moderate level of noise, which increases the minimum detectable feature size.^51^ The latter should therefore be estimated before the chemical measurement process, with a reasonable educated guess, based on domain knowledge, for example.

## Conclusion

In the present work, we utilized the concept of JND from psychophysics in a novel area of application, defining an easily calculated and interpretable metric for determining detection limits of measured analytes in imaging and mapping techniques. We have used LIBS and LA-ICP-MS elemental mappings for proof of principle, but the developments presented here can be used for any imaging technique. While the conventional LOD by IUPAC is very well-established in zero-dimensional contexts like simple univariate calibration methods using the regression line or methods employing signal-to-noise ratio calculations in one-dimensional data, we have shown in our work that LOD is sometimes not sensitive enough for discerning salient signals in two-dimensional data settings like imaging and mapping data. Since numerical values of signals in images are visually presented as different color shades, which is an additional layer of information not present in 0D and 1D settings, we have opted for an alternative metric, the JND, for determining detection limits. Contrary to LOD, JND incorporates more explicitly the nonlinear sensitivity of the human visual system to different color shades (gray levels in the present work), which allows for a finer detection of notable spatial features in images and lower absolute values of measured signals. We have shown that a simple combination of median filtering and JND map calculation can discern signals far below the LOD value in complex images of moderate noise from LIBS measurements. Although not explored in this work, detecting low-intensity signals in high noise is of particular importance in determining minor and trace elements in any mapping technique and in analysis of maps obtained with small laser beam sizes in methods like LIBS and LA-ICP-MS. In low-noise images like LA-ICP-MS maps presented here, the use of JND did not lead to substantial advantage over LOD in terms of lowering detection limits in concentration levels, but feature recognition was still comparably strong by both metrics. We emphasize that there are a number of potential improvements that could be considered in order to make the JND metric a more generally applicable figure of merit. Currently, the only independent variable explicitly taken into account is the absolute gray level of a selected background of an image under consideration, represented by the 95th percentile of the median-filtered background gray levels. However, there are other variables that influence the signal detectability irrespective of gray levels, namely, the expected size of an observable spatially resolved feature which is also correlated to the maximum acceptable beam size and the appearance of noise and structural patterns in the background. It is well-known that in higher noise levels, smaller features are more readily obscured than larger ones,^51^ so the feature size and background noise should ideally be treated independently of the gray levels. A more generalized JND metric could then report the lowest perceptible signal intensity given the measured background noise and expected feature size, possibly including additional variables such as local gradients in the background and correlations between the independent variables, making signal detection a multivariate problem. Additional complications may arise if the noise increases with signal (heteroscedasticity), as is a common occurrence in chemical measurements. While this will certainly increase the LOD value,^1^ the effect on JND is difficult to predict in advance and would probably depend on existing background patterns. As mentioned in the introduction, the theoretical approach applied in this work only to grayscale images could be extended to colored images with different colormaps in an arbitrary three-component color space. In an ideal perceptually uniform colormap, the JND is a fixed value, and any two colors would be considered visually distinguishable if the Euclidean distance between their coordinates is greater than the JND.^31,52^ In practice, many commonly used scientific colormaps are perceptually nonuniform, and some are unreadable for individuals with color-vision deficiencies.^52^ The use of such colormaps can introduce many additional complications, such as the appearance of false edges and visual artifacts.^53^ For these reasons, the discussion of color-related nuances was considered beyond the scope of this work. Given the importance and complexity of visual signal perception in the analysis and interpretation of imaging data, it is clear that additional theoretical developments are necessary, and work is in progress to find precise and practically interpretable extensions of the suggested imaging metric.

## Supplementary Material



## Notes

Calibration measurement data, details of the computational algorithm, application instructions (PDF)
