# Supplementary material for: Signal Perception in Two-Dimensional Mapping Techniques: Just Noticeable Difference as a Visual Limit of Detection
Source: Anal Chem. 2025 Sep 15;97(37):20108–16. doi: 10.1021/acs.analchem.5c02398 (PMC12461682; doi:10.1021/acs.analchem.5c02398)
Supplement: Supplementary file 1 [file ac5c02398_si_001.pdf]

SUPPORTING INFORMATION

Signal perception in two-dimensional mapping techniques - just noticeable difference as a visual limit of detection

Filip Cernatič,† Lukas Brunnbauer,‡ Kristina Mervič,§ Jakob Willner,‡ Andreas Limbeck,‡ Martin Šala† *

† National Institute of Chemistry, Department of Analytical Chemistry, SI-1000 Ljubljana, Slovenia;

‡ TU Wien, Institute of Chemical Tech-nologies and Analytics, AT-1060 Vienna, Austria;

§ National Institute of Chemistry, , Department of Catalysis and Chemical Reaction Engineering, SI-1000 Ljubljana

Table of contents

Figure S1. LIBS Fe and La maps with highlighted background selections for signal averaging.

Table S1. Average signal intensities from the rectangular portions of the largest circles in Figure S1.

Table S2. Slope and intercept values for the calibration lines in LA-ICP-MS two-point calibrations (obtained from NIST SRM 610 and 612 calibration measurements).

Figure S2. A schematic representation of the algorithm for LOD and JND calculation used in the application.

Figure S3. Application instructions, part 1.

Figure S4. Application instructions, part 2.

Figure S5. Application instructions, part 3.

Figure S6. Application instructions, part 4.

Figure S7. Application instructions, part 5.

Figure S8. Application instructions, part 6.

Figure S9. Application instructions, part 7.

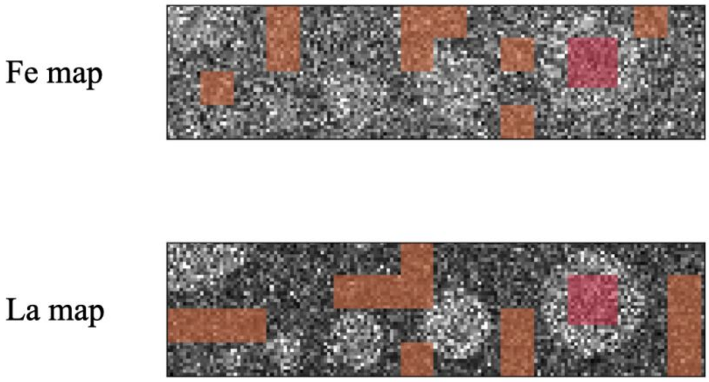

Figure S1. LIBS Fe and La maps with highlighted background selections (dark orange) and selected part of the largest circle for signal averaging (raspberry).

	**Fe map**	**La map**
Average signal intensity	49.896	18.748

Table S1. Average signal intensities ($\bar{x}$) from the rectangular portions of the largest circles in Figure S1. The calibration slopes for LIBS are then calculated as $(\bar{x} - \bar{x}_b)/c$ where $\bar{x}_b$ is the average intensity of the background (dark orange in figure 1) and c is the concentration of the element (in mass fraction).

	**Gd map**	**Ba map**
Slope (5µm beam)[µg/g]$^{-1}$	4.257	43.97
Intercept (5µm beam)	-134.2	-1525.0
Slope (10µm beam)[µg/g]$^{-1}$	45.08	249.9
Intercept (10µm beam)	-1546.6	-7959.1
Slope (20µm beam)[µg/g]$^{-1}$	117.37	579.4
Intercept (20µm beam)	-3497.7	-7763.1

Table S2. Slope and intercept values for the calibration lines in LA-ICP-MS two-point calibrations (obtained from NIST SRM 610 and 612 calibration measurements).

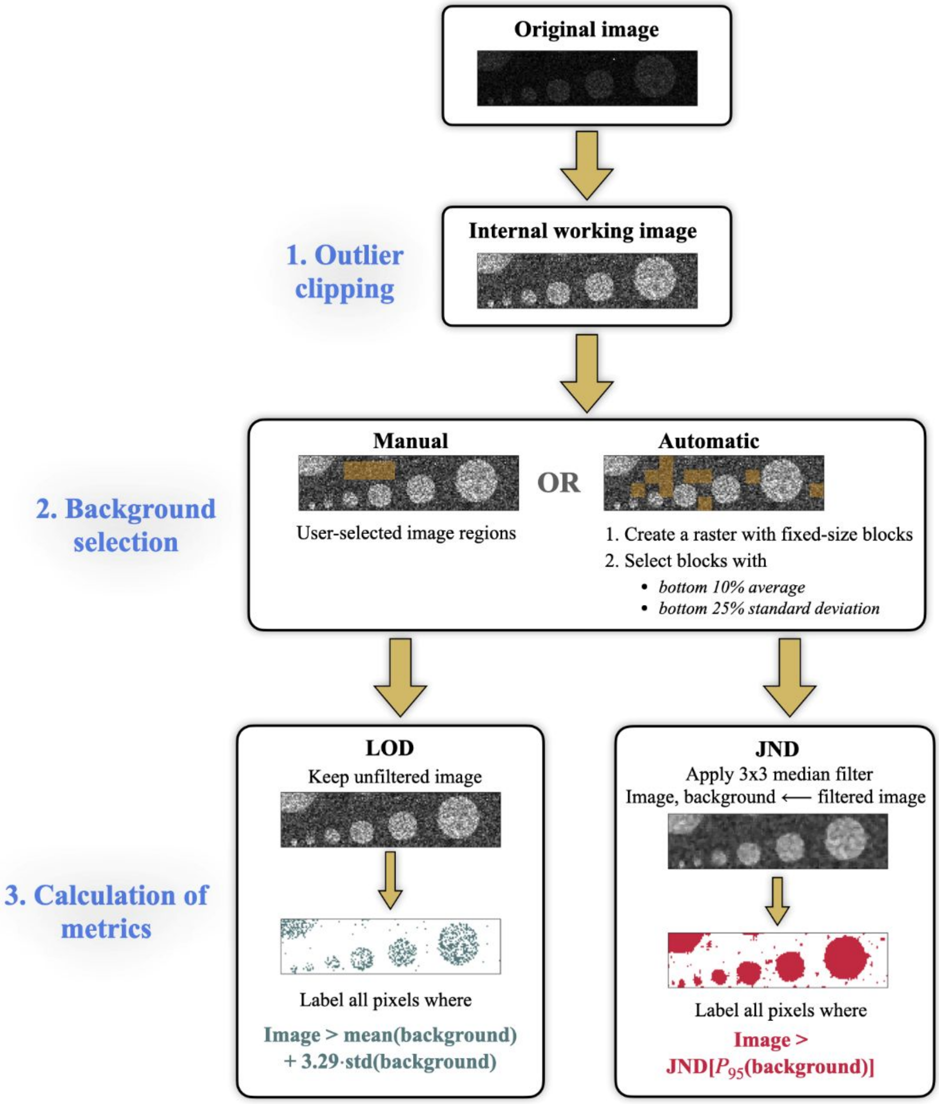

Figure S2. A schematic representation of the algorithm for LOD and JND calculation used in the application. The reader is referred to the next page for additional explanation on some of the steps.

Some details on different steps of the algorithm

Outlier clipping: The user chooses the desired percentile clipping level to suppress low and high outlier pixels. In the main part of our work, we use 0th percentile, and 99th percentile of the intensity distribution of an uploaded csv file to limit the data range. This implies that pixels with intensity levels above the 99th percentile, are set to the value of the 99th percentile.

Background selection: Background selection can be done either manually or automatically. While manual background selection allows for selecting rectangular blocks of any size and shape, in automatic background selection the blocks belonging to the raster are always square with size depending on image dimensions. For a given image with width W and height H, the raster block size for automatic background selection is calculated as follows,

$$\text{Block size (pixel units)} = \begin{cases} 10x10\,;\ if\ min(W,H)\ >= 40, \\ 5x5\,;\quad if\ min(W,H)\ >= 20, \\ 2x2\,;\ \ if\ min(W,H)\ <\ 20. \end{cases}$$

Images with one-pixel width or height either are automatically rejected by the application, hence 1x1, 2x1 or 1x2 block sizes are not included by the above function.

Calculation of metrics: For calculating LOD, the percentile-clipped image is used, while for calculating JND, the median-filtered image is used, which is stored in the browser when calculated during the application's session and (not accessible to the user). In both cases, if the automatic background selection is chosen, background blocks are always computed from the unfilltered image.

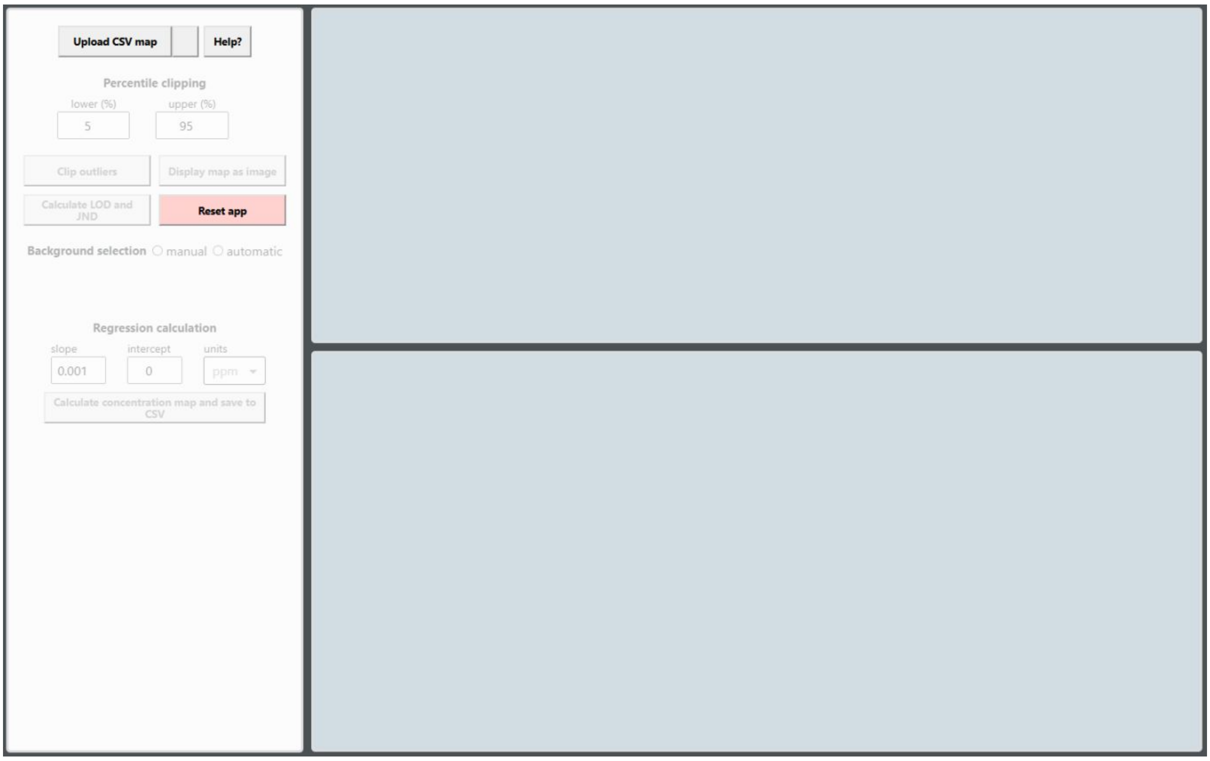

Figure S3. Application instructions, part 1: The initial user interface with the control panel (left gray area) and display area with two green panels to the right. Upon launching the application, three buttons in the control panel are initially enabled: »Upload CSV map«, »Help?« and »Reset app«. By clicking on the »Upoad CSV map« button, the user is prompted to choose a file of comma-separated values (CSV) format from their local computer (see Figure S4). »Help?« button contains detailed instructions on how to use the app. At any time, the user may click on »Reset app« button to reset the session to initial settings and clear all uploaded data.

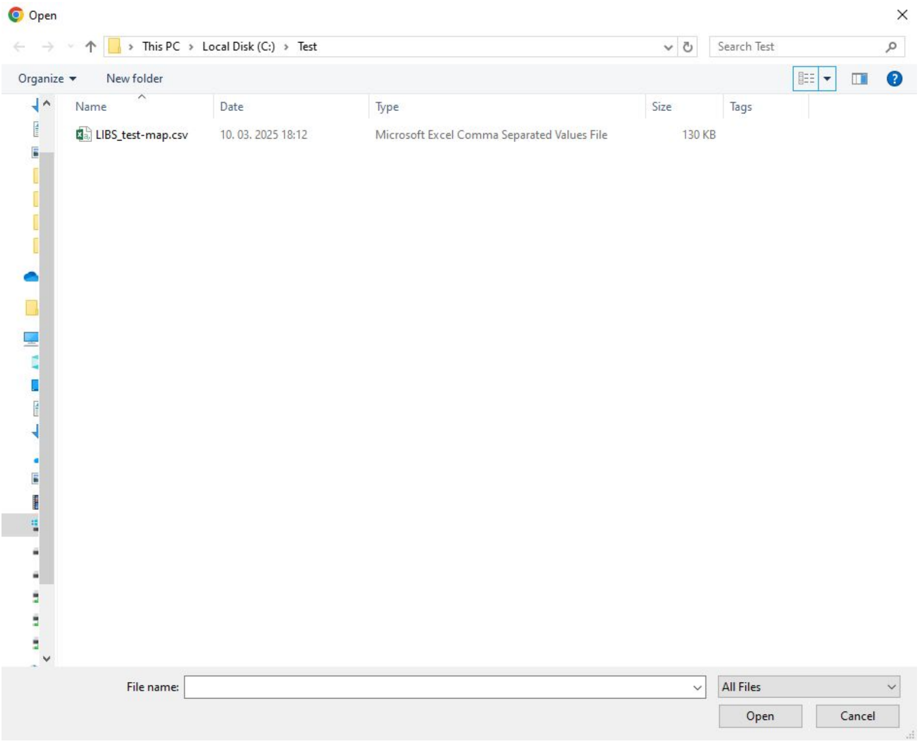

Figure S4. Application instructions, part 2: The file selection dialog, triggered after clicking the »Upload CSV map« button. The uploaded csv file should contain only decimal numbers, separated by the comma delimiter. Alphabetic characters, non-numerical symbols and other delimiters are not accepted.

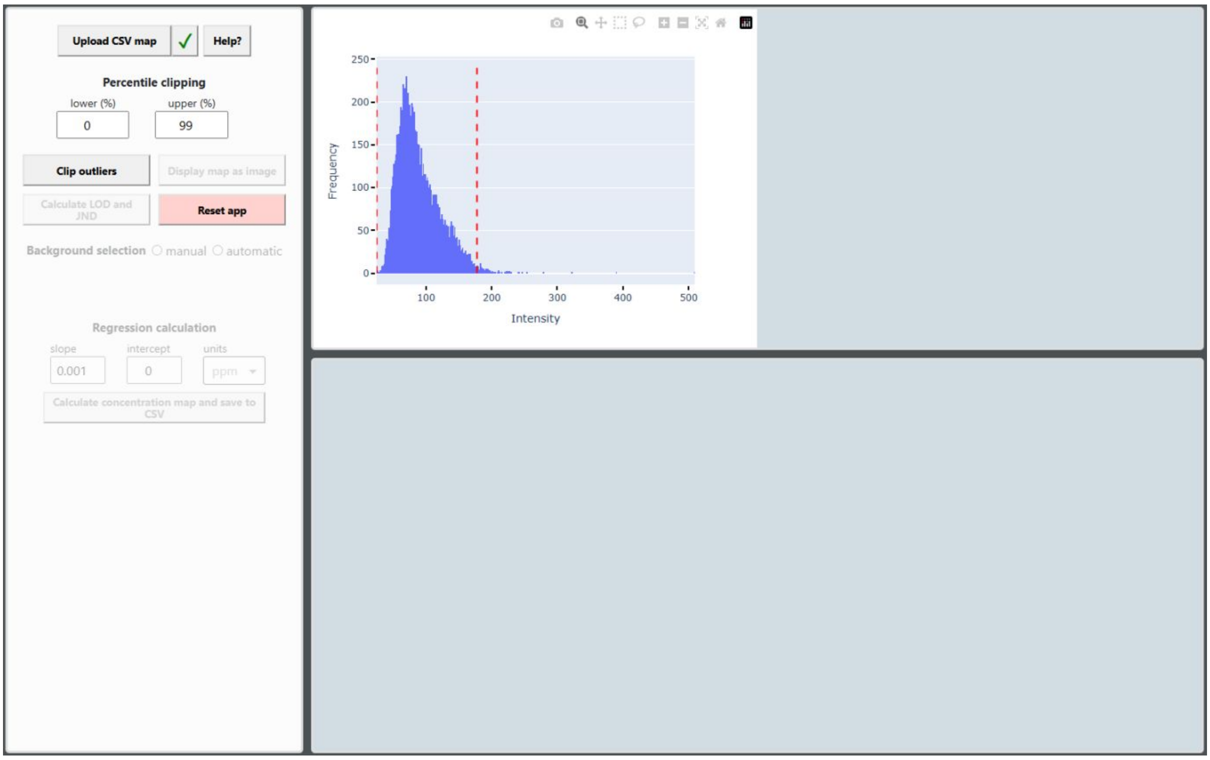

Figure S5. Application instructions, part 3: Upon uploading a CSV map with correctly formatted data (see Figure S4), a green tick mark (✓) will appear in the small box to the right of »Upload CSV map« button, and a histogram of intensity distribution from the CSV map will appear in the top left corner of the display area of the application. Attempting to upload an incorrectly formatted CSV file will produce a red cross mark (✗) in place of (✓). Additionally, the options for clipping outliers will become available, with two input boxes for specifying the lower and upper percentile of the intensity distributions. The percentile clipping works by removing low and high outliers by changing their values to the value of the lower and higher percentile, respectively, of the intensity distribution. The percentile locations are also dynamically displayed on the histogram as dashed red lines, for visual guidance.

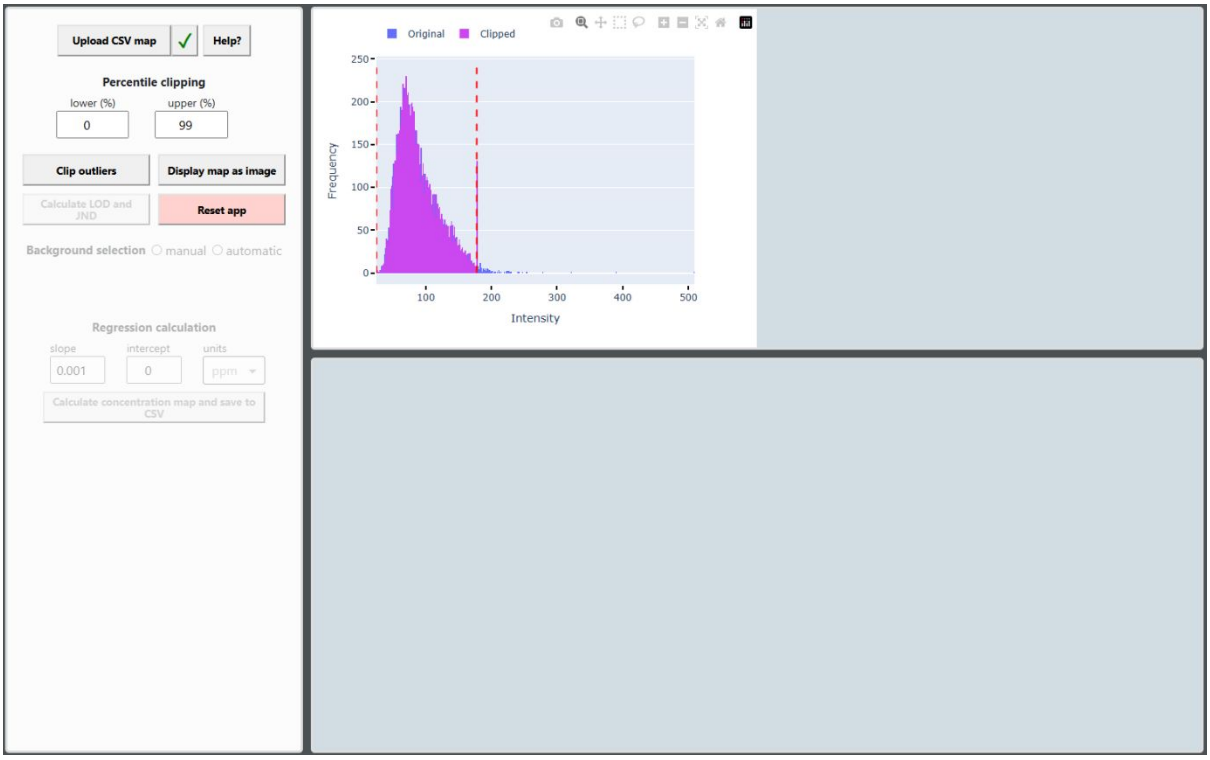

Figure S6. Application instructions, part 4: By clicking on the »Clip outliers« button, the intensity distribution is clipped and the modified percentile-clipped map is internally stored as an array for the duration of app's session. The original and percentile-clipped distribution are displayed on the same histogram for comparison. Then, the »Display map as image« button is enabled, which allows the user to visualize the modified map as a grayscale image. All subsequent calculations operate on the modified map.

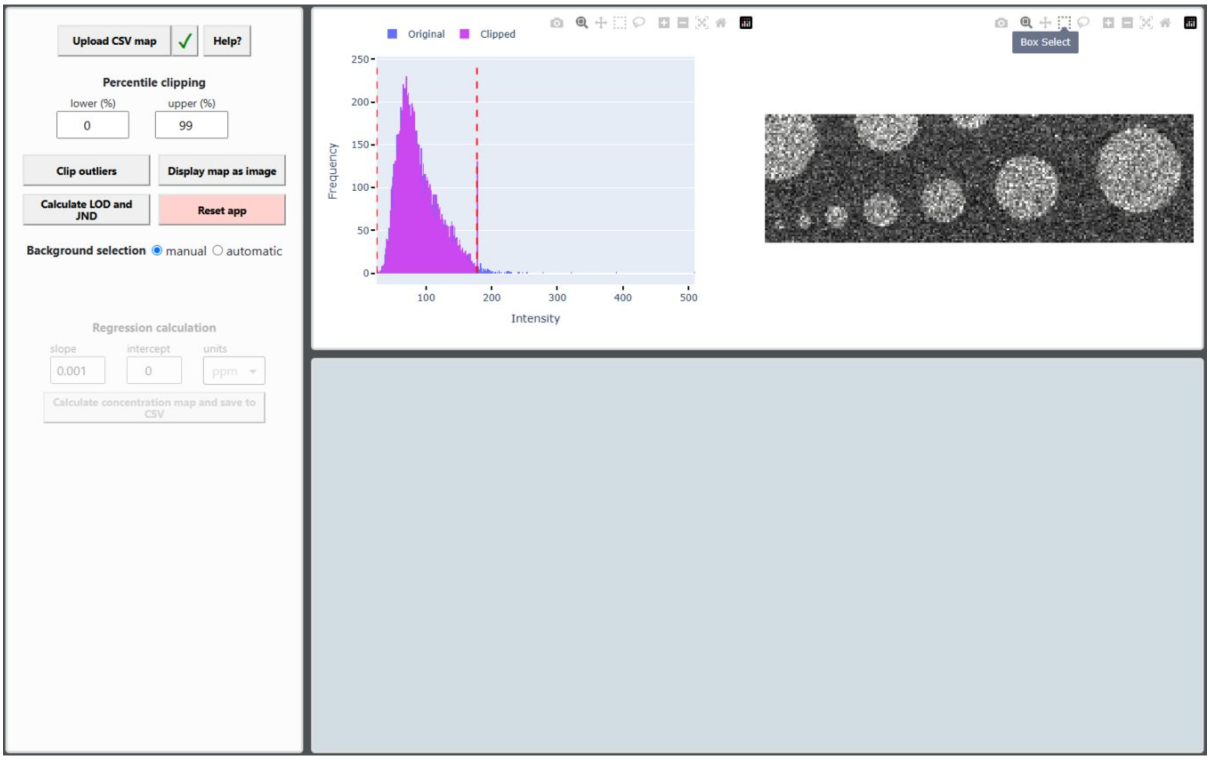

Figure S7. Application instructions, part 5: After displaying map as a grayscale image, which appears to the right of the histogram, the user may calculate LOD and JND metrics in intensity units by clicking on the »Calculate LOD and JND« button. For calculating the two metrics, estimating a background intensity distribution, ideally devoid of any signals, is necessary. Two options are available for this, either »manual« or »automatic« background selection, which can be chosen by clicking on the respective radio buttons in the control panel. The »manual« background selection is done by clicking on the »Box select« option in the top-right toolbar (to the top of the displayed image). On the other hand, »automatic« background selection is done internally by the app as detailed above.

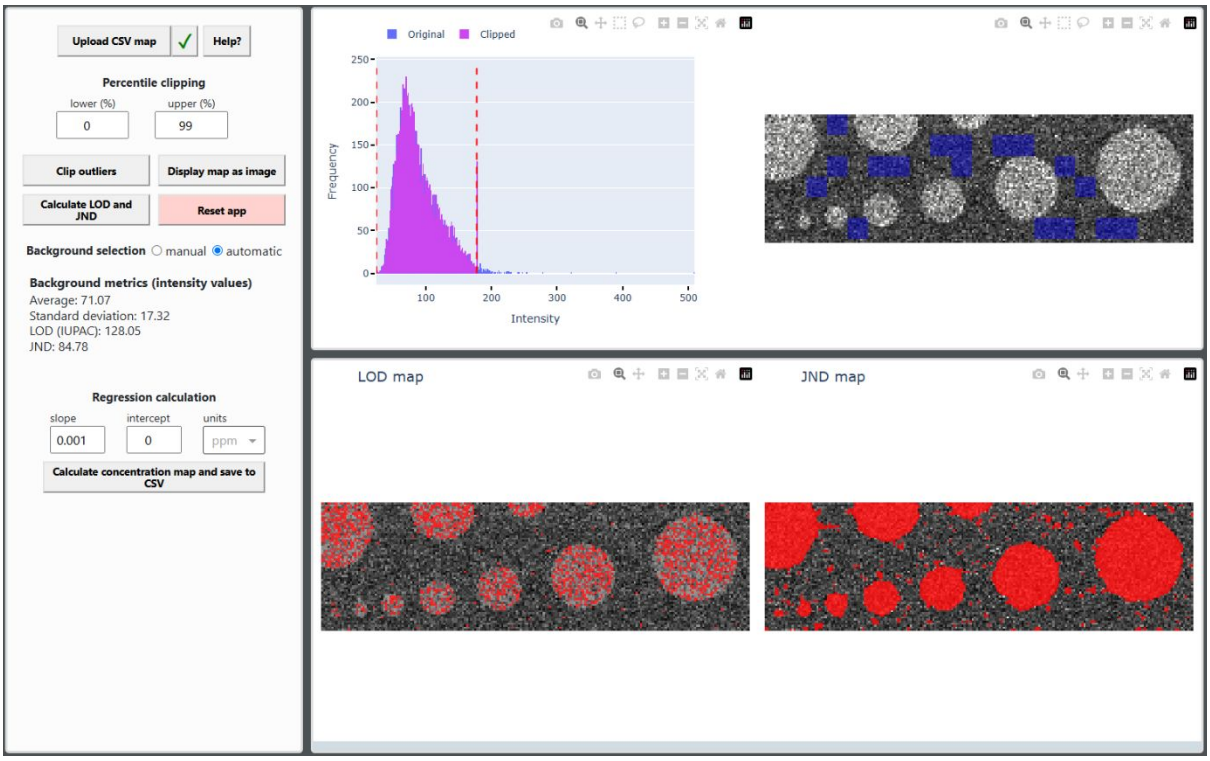

Figure S8. Application instructions, part 6: After calculating the LOD and JND, their values in intensity units are printed out in the control panel, in addition to the average and standard deviation of the background. Two more images, the LOD and JND maps, also appear in the bottom panel of the display area. On these maps, pixels that exceed the LOD and JND, respectively, are labelled with red. In addition, regions which were selected for background estimation, are highlighted in blue in the image (top-right panel) after the calculation of LOD and JND is finished.

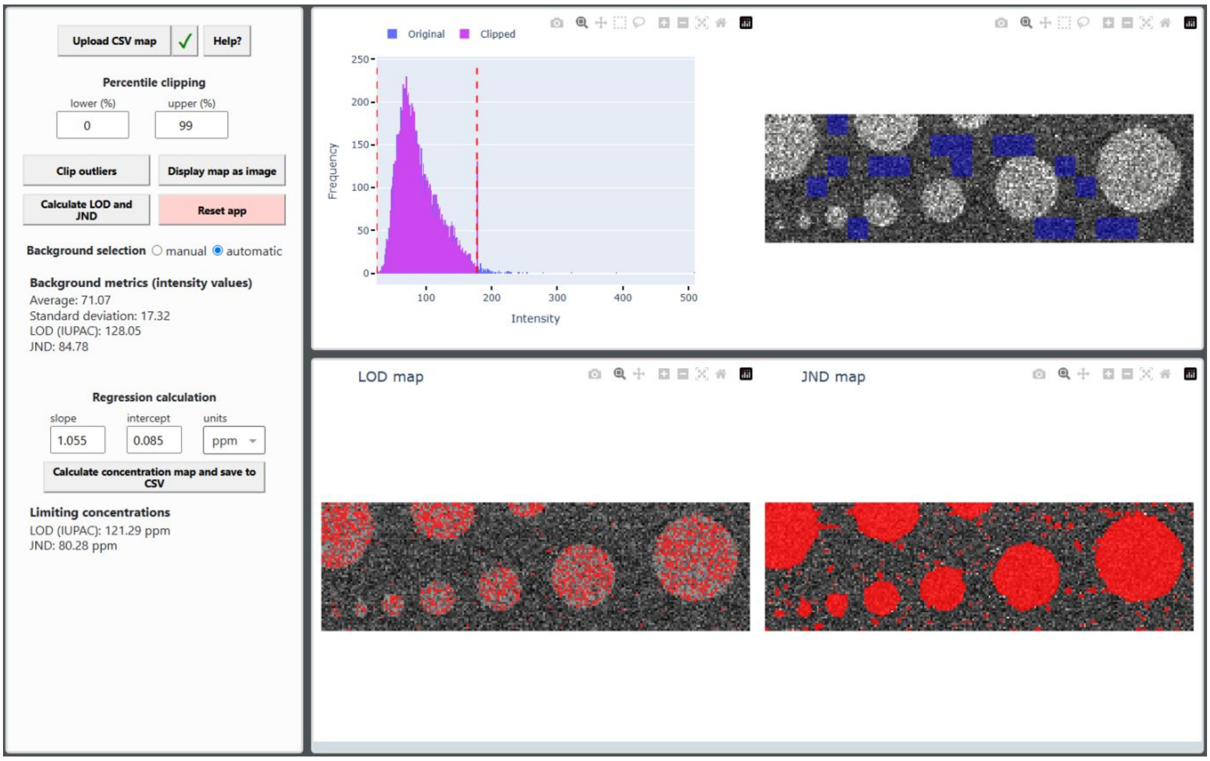

Figure S9. Application instructions, part 7: Finally, upon calculating the LOD and JND, the »Regression calculation« options are enabled, which allow the user to convert the original (percentile-clipped) elemental map from intensity units to concentration units. The available parameters are »slope« and »intercept« for the calibration line, and »units«, which are specified for reporting purposes. The available units are ppm, ppb, ppt, %w/w, µg/g and mg/g. After specifying all the parameters and clicking on the »Calculate concentration map and save to CSV«, the elemental map in concentration units is saved to the download destination specified by the browser as »[uploaded_csv_name]_calibrated.csv«. In addition, the LOD and JND are displayed in concentration units at the bottom of the control panel.
